# Genetic Diversity Revealed by Single Nucleotide Polymorphism Markers in a Worldwide Germplasm Collection of Durum Wheat

**DOI:** 10.3390/ijms14047061

**Published:** 2013-03-28

**Authors:** Jing Ren, Daokun Sun, Liang Chen, Frank M. You, Jirui Wang, Yunliang Peng, Eviatar Nevo, Dongfa Sun, Ming-Cheng Luo, Junhua Peng

**Affiliations:** 1 Key Laboratory of Plant Germplasm Enhancement and Specialty Agriculture, Chinese Academy of Sciences, Wuhan 430074, Hubei, China; E-Mails: renjing0@gmail.com (J.R.); sundaokun1@gmail.com (D.S.); chenliang1034@126.com (L.C.); 2 Graduate University of Chinese Academy of Sciences, Beijing 100049, China; 3 Department of Plant Sciences, University of California, Davis, CA 95616, USA; E-Mails: frank.you@agr.gc.ca (F.M.Y.); wangjirui@gmail.com (J.W.); 4 Cereal Research Centre, Agriculture and Agri-Food Canada, Winnipeg, MB R3T 2M9, Canada; 5 Institute of Plant Protection, Sichuan Academy of Agricultural Sciences, Chengdu 610066, Sichuan, China; E-Mail: pengyunliang@yahoo.com.cn; 6 Institute of Evolution, University of Haifa, Mount Carmel, Haifa 31905, Israel; E-Mail: nevo@research.haifa.ac.il; 7 College of Plant Science & Technology, Huazhong Agricultural University, Wuhan 430071, Hubei, China; E-Mail: sundongfa@mail.hzau.edu.cn; 8 Department of Soil and Crop Sciences, Colorado State University, Fort Collins, CO 80523, USA

**Keywords:** *T. durum*, landrace, cultivars, molecular marker, SNP, genetic structure

## Abstract

Evaluation of genetic diversity and genetic structure in crops has important implications for plant breeding programs and the conservation of genetic resources. Newly developed single nucleotide polymorphism (SNP) markers are effective in detecting genetic diversity. In the present study, a worldwide durum wheat collection consisting of 150 accessions was used. Genetic diversity and genetic structure were investigated using 946 polymorphic SNP markers covering the whole genome of tetraploid wheat. Genetic structure was greatly impacted by multiple factors, such as environmental conditions, breeding methods reflected by release periods of varieties, and gene flows via human activities. A loss of genetic diversity was observed from landraces and old cultivars to the modern cultivars released during periods of the Early Green Revolution, but an increase in cultivars released during the Post Green Revolution. Furthermore, a comparative analysis of genetic diversity among the 10 mega ecogeographical regions indicated that South America, North America, and Europe possessed the richest genetic variability, while the Middle East showed moderate levels of genetic diversity.

## 1. Introduction

Modern wheat cultivars usually refer to two species: hexaploid bread wheat, *Triticum aestivum* (2*n* = 6*X* = 42, AABBDD), and tetraploid, hard or durum-type wheat, *T. durum* (2*n* = 4*X* = 28, AABB) [1]. Durum wheat is traditionally grown around the Mediterranean Sea and is the most common cultivated form of allotetraploid wheat. Currently, more than half of the durum wheat is still grown in the Mediterranean basin, mainly in Italy, Spain, France, Greece, West Asian, and North African countries [2].

Wheat domestication took place 12,000 years ago in the Near East, with the wild ancestor (*T. dicoccoides*) giving rise to the first domesticated form (emmer wheat, *T. dicoccum*) [3]. About 2000 years after this event, durum wheat, which is characterized by free threshing, appeared in the eastern Mediterranean and replaced its ancestor *T. dicoccum* to become the major cultivated form of allotetraploid wheat by the second millennium BC [3–5]. Durum was part of the initial crop package introduced into Europe and North Africa during the Neolithic period but was preferred in the western Mediterranean basin [6], whereas emmer was the staple crop in Ancient Egypt until the introduction of durum in the Hellenistic Period [7]. Durum wheat continued to spread throughout Europe at the end of the 15th century [8]. That is, when Europeans first touched the shores of the Americas across the Atlantic in 1492, the Columbian Exchange (artificial re-establishment of connections through the commingling of Old and New World plants, animals, and bacteria.) allowed durum wheat from the Old World to the New World [9,10]. Especially in the Spanish colonial periods during the 16–17th centuries, European agriculture had a profound effect on the Americas. The most recent history of durum wheat has been marked by modern genetic improvement, involving the replacement of landraces by inbred varieties and the introduction of dwarfing genes (second part of the 20th century) [3]. These historical events are likely to have altered the original genetic structure and genetic diversity pattern of wheat.

Molecular markers are particularly useful for the evaluation of genetic diversity in wheat and other crop species with a narrow genetic base [11]. To date, a variety of molecular marker techniques are available for genome analysis in wheat. Molecular markers that did not rely on genomic sequence information were designed first, including restriction fragment length polymorphisms (RFLPs) [12–14], random amplified polymorphic DNA (RAPD) [14–16], and amplified fragment length polymorphism (AFLP) [11,14,17–20]. These markers have been used successfully for genetic mapping, phylogenetic relationships [17,18], comparative genomic studies [20], and diversity evaluation [18,19]. However, none of them have been used extensively in breeding programs because they do not meet the requirements for efficient application in marker-assisted-selection (MAS), *i.e.*, adaptability to flexible and high-throughput detection methods, high efficiency with low-quantity and low-quality DNA, low-cost per assay, tight linkage to target loci, and the high level of polymorphism in breeding materials [21,22].

Until now, simple sequence repeat (SSR) markers relying on genomic sequences have been proven to be the most widely used DNA marker type in characterizing germplasm collections of crops, because of their easy use, relatively low cost, and high degree of polymorphism provided by the large number of alleles per locus [23,24]. In the past decade, thousands of SSR markers have been developed for wheat and more than 4000 have been mapped genetically (see GrainGenes: A Database for Triticeae and *Avena.*[25]). However, operationally, there have been problems in their use caused by challenges in accurately sizing SSR alleles due to PCR and electrophoresis artifacts [26].

More recently, single nucleotide polymorphism (SNP) markers gained significant attention because they are bi-allelic in nature and occur at a much higher frequency in the genome than SSRs and other markers. Furthermore, their genotyping can be easily automated [26]. In crops, the availability of SNP genotyping platforms would facilitate the genetic dissection of traits of economic importance and the application of marker-assisted and genomic selection [21,27–29]. Moreover, SNPs are the most abundant class of sequence variability in the genome and thus have the potential to provide the highest map resolution [26,30]. Genome-wide maps comprised of large numbers of SNP markers have been reported in *Arabidopsis*[31], rice [32], soybean [33], and barley [34]. However, so far only a limited number of SNPs has been reported in wheat [35–40], because large-scale SNP discovery in wheat is limited by both the polyploidy nature of the organism and the high sequence similarity found among the three homoeologous wheat genomes [38,41]. Also, none have been reported on genetic diversity and genetic structure detected by SNP markers in world-wide durum wheat germplasm resources.

Information about the genetic diversity and genetic structure in germplasm is of fundamental importance for crop improvement [24]. It is widely argued that the genetic diversity of major crops, especially self-pollinating cereals, has suffered an overall reduction with time, due to the pressure of pure-line selection applied in breeding programs [42–44]. Genetic diversity in durum wheat germplasm were studied using several types of molecular markers. However, SNP-detected diversity pattern and genetic relationships in a worldwide germplasm collection of durum wheat have not been reported. Herein, the objectives of our study were to (a) evaluate the genetic diversity in a global durum wheat collection using SNP markers covering the whole genome; (b) unravel the genetic structure of durum wheat; and (c) assess genetic variation temporally and spatially by comparing the diversity among released periods of varieties and among different geographical origins, respectively.

## 2. Results

### 2.1. SNP Marker Quality and Genomic Distribution

A total of 230,400 data points were generated by genotyping of 150 durum wheat accessions with multiplexed 1536 Illumina Golden Gate SNP assay. Out of 1536 SNPs presented in our oligonucleotide pool assay (OPA), 1366 (89%) SNPs with high quality genotype calls were obtained, while the other 10% failing to generate clear genotype clustering were removed. Out of the 1366 scoreable SNP markers, 420 were monomorphic across all the 150 accessions and the overall polymorphism rate was 69.3%. Because SNP markers are mainly bi-allelic, therefore, all SNPs showed two alleles only. The 946 polymorphic SNPs markers were used for further analysis. Marker distribution, Nei’s gene diversity, and PIC values estimated for each chromosome and genome were listed in Table 1.

SNPs loci were not evenly distributed across the seven homoeologous groups, and coverage ranged from 108 in group 5 to 161 loci in group 6. Nei’s gene diversity and PIC values across groups ranged from 0.2004 to 0.2508 and from 0.1656 to 0.2006, respectively. The chromosome group 1 had higher genetic diversity and the group 3, 4 and 5 had lower genetic diversity than the genome-wide average (Table 1).

Of the polymorphic loci, 516 and 430 were located in A and B genomes of durum wheat, respectively. As shown in Table 1, a higher genetic diversity was detected in genome B with Nei’s gene diversity, and PIC values of 0.2384 and 0.1970, respectively, while 0.2193 and 0.1819 for genomes A, respectively. This difference between genome A and B was not statistically significant for both gene diversity (*t* = 1.459, *p* = 0.195, paired *t* test) and PIC (*t* = 1.488, *p* = 0.187, paired *t* test). In the A genome of durum wheat, chromosome 6A had higher genetic diversity (Nei’s gene diversity, 0.2526; PIC, 0.2072), and chromosome 4A had lower genetic diversity (Nei’s gene diversity, 0.1899; PIC, 0.1576) than the rest of chromosomes (Table 1). In the B genome, genetic diversity was lower in chromosome 4B and 5B than the genome-wide average, while genetic diversity was higher in chromosome 1B (Nei’s gene diversity, 0.2695; PIC, 0.225) than the genome-wide average (Table 1).

### 2.2. Genetic Structure

**Figure 1:**
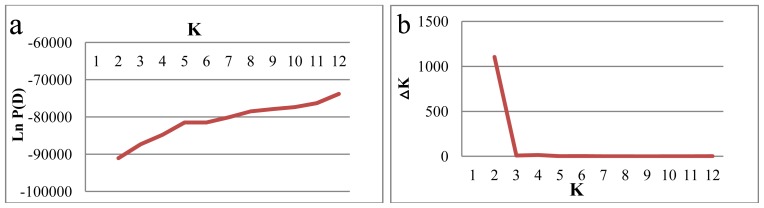
Estimation of the most probable number of clusters (*K*), based on five independent runs and *K* ranging from 1 to 12. (**a**) Evolution of the natural logarithm probability of the data against *K*; and (**b**) Magnitude of *ΔK* for each *K* value.

**Figure 2:**
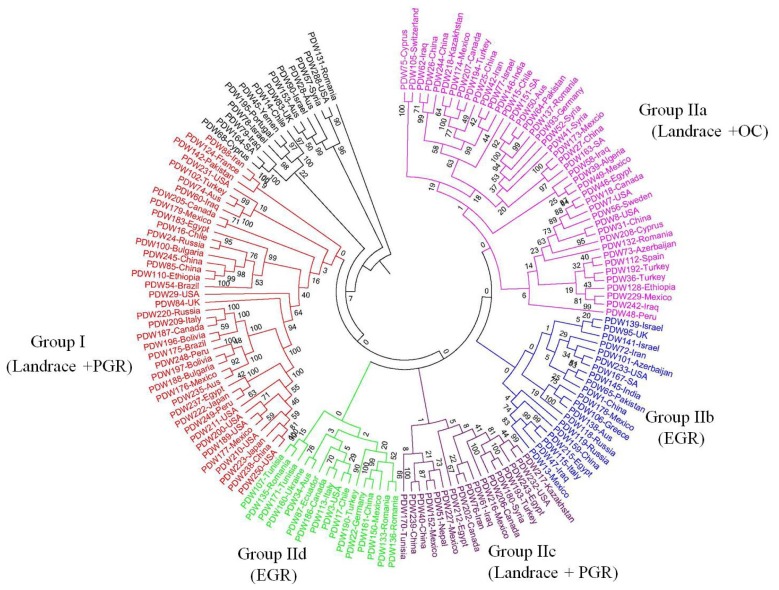
Dendrogram of 150 *T. durum* accessions based on the shared-allele genetic distance calculated from data of 946 SNP markers, using the NJ algorithm as the clustering method. Numbers on nodes are bootstrap probabilities estimated by permutation test with 1000 replications.

Genotyping data generated by the 946 polymorphic SNP markers were used for genetic structure analysis, using the Bayesian clustering model implemented in the Structure software. The estimated log probability of the data (*Ln*P(D)) increased continuously with increasing *K* and there was no obvious *K* value clearly defining the number of populations (Figure 1a). However, the rate of change in the Napierian logarithm probability relative to standard deviation (*ΔK*) [45] suggested that the best structure was *K* = 2 (Figure 1b). Thus, the analyzed durum wheat germplasm can be divided into two genetically distinct groups. Similarly, the unrooted NJ tree based on shared-allele genetic distances also distinguished two major groups of accessions (Groups I, II), corresponding to the structure grouping (Figure 2). However, group II can be further divided into four subgroups: IIa, IIb, IIc, and IId. Ecogeographical origin, improvement status (landraces *vs*. cultivars), and pedigree information of accessions were analyzed to explain the inferred structure.

Group I contained 39 accessions, about half (20/39) of which were collected from the Americas (North America and South America). Further analysis of these accessions showed that this group is dominated by landraces (16) and cultivars released during the Post Green Revolution (PGR) (14) (Figure 2).

Group II contained 96 accessions, which can be further divided into four big subgroups: IIa, IIb, IIc, and IId. Although the grouping pattern is very ecogeographically heterogeneous in each subgroup, the grouping pattern of some accessions appeared to be associated, to some extent, with the release period of varieties (Figure 2). Group IIa is dominated by landraces and old cultivars (OC). Group IIc is dominated by landraces and cultivars released during the Post Green Revolution. Both group IIb and IId are dominated by cultivars released during the Early Green Revolution (EGR).

### 2.3. Genetic Diversity between Landraces and Cultivars

As shown in Table 2, difference between landrace and cultivar was significant for Nei’s gene diversity (*t* = 7.214, *p* < 0.001, paired *t* test) and PIC (*t* = 9.026, *p* < 0.001, paired *t* test). The higher genetic diversity was detected using SNP markers in the cultivars with Nei’s gene diversity and PIC values of 0.2310 and 0.1919, compared to 0.2192 and 0.1800 for the landrace, respectively. Furthermore, molecular variance component in cultivars and landraces was compared to serve as a complementary indicator for genetic diversity. Analysis of molecular variance (AMOVA) revealed that individuals within cultivars (65.54%) are highly genetically variation in relation to individuals within landraces (33.97%) (Table 3). Similarly, the higher polymorphic level obtained from the cultivars also reflect greater genetic variation compared to that in the landraces. Of the 946 polymorphic SNP markers over the panel of 150 accessions, 756 showed polymorphism (756/946 = 79.9%) among the 53 landraces, while 933 showed polymorphism (933/946 = 98.6%) among the 97 cultivars (Table 2). The panel of 53 landraces has a significant lower level of genetic diversity than the panel of 97 durum wheat cultivars. But previous research showed that landraces had wide genetic diversity, while the cultivars had narrow genetic diversity due to high selection pressure and genetic drift in breeding programs [20,46,47].

In order to explain the reasons why the higher level of genetic diversity exists within improved accessions, the 97 cultivars were further divided into three temporal groups: OC, EGR and PGR. As shown in Table 2, a loss of genetic diversity was observed from OC to EGR (Nei’s gene diversity, *t* = 6.484, *p* < 0.001, paired *t* test; PIC, *t* = 6.304, *p* < 0.001, paired *t* test), but an increase in PGR was observed (Nei’s gene diversity, *t* = 9.617, *p* < 0.001, paired *t* test; PIC, *t* = 9.885, *p* < 0.001, paired *t* test). That is, genetic diversity was narrowed down from 1930 to 1980, but enhanced from 1981 to 2009.

Noteworthy, plant height, as an extremely important target trait in modern wheat breeding, also showed significant variation/decrease. The “Green Revolution” in cereals was achieved by reducing plant height, thereby reducing lodging susceptibility and increasing grain yield [1,48]. As shown in Table 4, mean plant height of landrace and old cultivars were 132.46 and 130.72, respectively, while cultivars released during the periods of EGR and PGR had a significantly lower plant height (*F* = 19.02, *p* < 0.01, ANOVA), with an average of 119.13 and 101.91, respectively.

### 2.4. Divergence between Landraces and Cultivars

**Figure 3:**
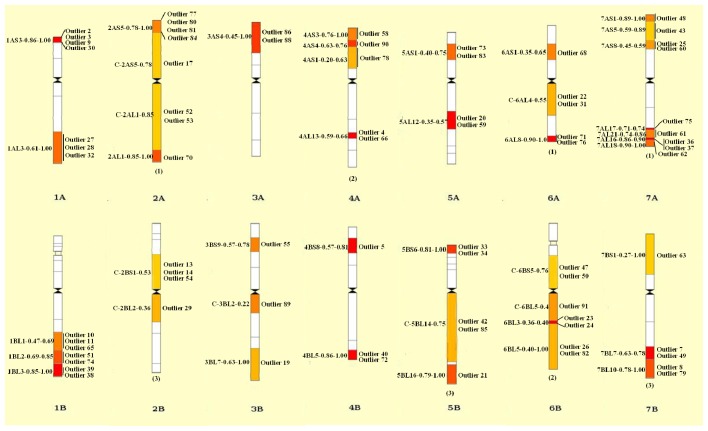
Chromosomal distribution of 92 outlier loci under positive selection. The codes of mapped loci are shown on the right of each chromosome and the intervals are indicated on the left. Details of codes are presented in Table 4. The number in parentheses at the bottom of each chromosome is the number of EST loci mapped in that chromosome without knowing the exact bin. Only those bins with mapped loci are indicated.

We conducted further analyses to identify candidate loci that are under positive selection between landraces and cultivars. An analysis of *Fst* on a locus-by-locus basis provided a cutoff for identifying loci that may be under positive selection [49]. Therefore, we used an outlier detection method implemented in the LOSITAN program [50]. Between landraces and cultivars, a total of 92 outlier loci under positive selection were identified. Chromosomal distributions of these loci were shown using wheat chromosome bin maps in Figure 3. A high portion of these loci (54.3%) was derived from chromosomes 2, 6, and 7. Among the 92 loci, P-EA (phosphoethanolamine methyltransferase), TsPAP1 (prolyl aminopeptidase 1), CPK10 (Calcium-dependent protein kinase), PI-PLC1 (phosphoinositide-specific phospholipase C1), RSZ38 (alternative splicing regulator), PDS (phytoenedesaturase), and LOX3 (lipoxygenase) gene, which play important roles in plant responses to biotic and abiotic stresses or in grain storage in wheat, were identified as under positive selection between landraces and cultivars. We inferred putative functions of these loci based on comparison to a protein sequence database (Table 5).

### 2.5. Genetic Diversity *vs*. Place of Origin

Knowledge of genetic diversity from different ecogeographic areas was expected to have a significant impact on the conservation and utilization programs of durum germplasm, allowing breeders to develop strategies to incorporate useful diversity in their breeding programs. A summary of the genetic diversity data of the 10 mega ecogeographical regions was shown in Table 6. Accessions in South America showed the highest values of both Nei’s gene diversity (0.2518) and PIC (0.2044), followed by North America (0.2351, 0.1937) and Western Europe (0.2299, 0.1902). On the contrary, the lowest level of Nei’s gene diversity and PIC were detected in South Asia (0.1575, 0.1258) and South Africa (0.1591, 0.1255). The remaining regions had a moderate level of Nei’s gene diversity and PIC value including the Middle East (0.1906, 0.1549), North Africa (0.2054, 0.1682), Oceania (0.2179, 0.1747), East Asia (0.2220, 0.1798), and East Europe (0.2183, 0.1792) (Table 6).

## 3. Discussion

### 3.1. SNP-Based Polymorphism and Genetic Diversity

Average Nei’s gene diversity and PIC values revealed by SNP markers in this study were 0.2280 and 0.1888, respectively (Table 1). Compared to the previous studies on durum wheat, this level of genetic diversity is not high. Moragues *et al*. [8] reported genetic diversity of 63 durum wheat landraces from the Mediterranean basin, and obtained PIC values of 0.24 and 0.70 for AFLP and SSR, respectively. Maccaferri *et al*. [2] studied genetic diversity of the elite durum wheat germplasm from Italy and other Mediterranean countries using SSR markers, and estimated a mean diversity index (DI) of 0.56. Relatively lower genetic variation revealed by SNP marker is an expected. Because SNP markers are mainly bi-allelic, the gene diversity and PIC thus cannot exceed 0.50, whereas the maximum can approach 1 for multi-allelic markers, such as SSRs.

Despite this fact, a sufficient level of genetic variation and similar variation trend can be detected using SNP markers. For example, our results are in agreement with previous studies that chromosomes 4A and 4B have relatively low genetic diversity due to the evolutionary translocation events involving chromosome 4A [14,51,52]. The greater genetic variation in the B genome than in the A genome was detected in this study (Table 1), which suggested a larger contribution of the B than A genome to durum genetic variation. The different contribution of A, B genomes to genetic variation was also demonstrated in previous studies by the use of SSRs [53], RFLPs [54] and AFLP [14] in common hexaploid wheat as well as in *T. dicoccoides*[1,55]. These results suggest that SNP can be used as an effective type of molecular markers for genetic evaluation in wheat.

### 3.2. Genetic Structure Raveled by SNP Markers

Genetic structure is similar among the 150 *T. durum* accessions, based on the Bayesian clustering model implemented in the Structure software and NJ algorithm implemented in POWERMARKER Ver. 3.25 and PHYLIP (Figures 1 and 2). Neither geographical nor ecological evidence for most accessions was detected in the grouping. This result suggested that the relationships we have found between countries are greatly affected by the within-countries variability. Consequently, countries that showed a large variability do not group easily (their grouping distance is large). AMOVA indicated that 90.81% of the genetic variation resided among accessions within the country (data not shown).

The reason might be that the gene flows via germplasm exchanges among different regions occurred frequently or that human transfer of genes in history made a very big admixture. This is consistent with the known history. Contact between the Old and New World after Columbus’ voyages allowed the exchange of many domesticated plants, including wheat. Especially, in the case of the Spanish colonies in Americas, it is well known that Spaniards not only tried by all possible means to introduce their own European culture, but also, with tenacity, to introduce many crops (including durum wheat landraces and cultivars) from Europe to the American territories [10]. Besides, emigration had a profound influence on the world in the 18th, 19th, and 20th centuries. Through trade routes and immigration, new varieties of wheat were sold or shared by people from different regions. Our ongoing experiment, including many more durum landraces collected from Spain and Mexico, will help us further understand germplasm exchanges between the Old and New World.

An alternative or complementary possibility may be found in breeding history. In this study, most of the accessions selected were cultivars (97/150 = 64.7%), and cultivars experienced primarily artificial selection, and only secondarily natural selection, for certain desirable characteristics. For example, breeding efforts focused on early maturity and yield increase before 1930, disease resistance from 1930 to 1970, and multiple disease resistance and quality improvement after 1970 [56–58]. Such human activities must have played a great part in a genetic shift. That is also why the grouping pattern of durum wheat accessions appeared to be associated with the released period of varieties to some extent (Figure 2).

However, not all accessions released from the same period were clustered in the same group. In contrast, some of accessions from the same geographic region were clustered together though into different groups corresponding to their geographical regions of collection (Figure 2). For example, South America contained 12 accessions; most of which (7/12) were clustered together into Group I, and others were mainly distributed in Group IId. Most of the American accessions (7/13) were clustered together into Group I. These results indicate that many of the accessions were clustered into groups corresponding to their geographical regions of collection, which may be due to the same environmental conditions or to agronomical practices.

Above all, such genetic structures and grouping patterns of the 150 durum wheat accessions were obviously affected by environmental conditions, release period of varieties, and gene flows via germplasm exchanges or artificial transfer of genes.

### 3.3. Genetic Diversity

Measurements of genetic diversity in crops have important implications for plant breeding programs and the conservation of genetic resources. In the present study, temporal and spatial genetic variation was analyzed by comparing the diversity among released periods of varieties and among different geographical origins, respectively.

#### 3.3.1. Temporally: Genetic Diversity *vs*. Year of Release

It has been argued that the level of genetic diversity in the modern durum wheat cultivar germplasm may have declined due to high-pure breeding selection pressure applied in breeding programs. This is also true for wild emmer wheat and wild barley due to global warming as discovered in a recent study by Nevo *et al*. [59]. However, our results demonstrated that there still existed a substantial level of genetic variation within a set of durum wheat cultivars as detected by SNP markers (Table 2).

We did find a significant reduction in the diversity of varieties released in the 1960s and 1970s, compared with the diversity levels in the landraces and old cultivars (1930–1964) (*p* < 0.001, paired *t* test). But the diversity was significantly increased in varieties released after the 1960s and 1970s (*p* < 0.001, paired *t* test) (Table 2). That is, genetic basis of durum wheat was narrowed down from 1930 to 1980, but was widened from 1981 to 2009 (Table 2). These results are in agreement with the previous reports by Soleimani *et al*. [11] and Maccaferri *et al*. [2]. Genetic diversity estimates in modern cultivars of durum wheat using AFLP and pedigree-based techniques showed that the level of genetic variation within the most recently developed cultivars is fairly substantial [11]. Likewise, microsatellite analysis also reveals a progressive widening of the genetic basis in the elite durum wheat germplasm [2]. However, we showed opposite results with Fu *et al*. who concluded genome-wide reduction of genetic diversity in Canadian wheat breeding programs [56–58]. The reasons may be due to differences in materials used and regions of collection. A worldwide durum wheat collection consisting of 150 accessions was used to estimate the genetic diversity in this study, while 75 Canadian hard red spring wheat (*T. aestivum* L.) cultivars were used in Fu’s study.

The low diversity levels of varieties released in 1965–1980 might be due to the “Early Green Revolution”, which was characterized by breeding semi-dwarf varieties possessing a higher yielding potential [60,61]. Interestingly, this deduction of genetic diversity was in agreement with decrease of plant height in durum wheat (Tables 2 and 4). The increase in genetic diversity from the 1980s may be explained by a change in the breeding strategy of the International Maize and Wheat Improvement Center (CIMMYT) in the late 1970s. During the last 50 years, CIMMYT has played a great role in wheat improvement including durum. Out of 140 durum varieties released from the period 1966–1992, 90 varieties (64%) are from CIMMYT crosses [62]. When CIMMYT realized the danger of narrowing down their germplasm base in the late 1970s, they changed the breeding strategy, aiming at increasing productivity while ensuring genetic diversity. Our result showed that genetic diversity was narrowed down from 1930 to 1980 but was enhanced from 1981 to 2009 (Table 2), indicating that CIMMYT breeders successfully increased the genetic diversity. The increase in genetic diversity can be obtained mainly through the introgression of various novel wheat materials [63,64], which can be proved in this study. Many cultivars used in this study were obtained by crossing *T. dicoccoides* and durum wheat. The pedigree information of these accessions used can be obtained from the Germplasm Resources Information Network (GRIN) [65] based on accession identifier # (Table 7).

Above all, the reason why genetic diversity is larger in cultivars than in landraces may be due to breeding strategy and breeders’ efforts. Alternatively, imbalanced sample size in the two groups (53 landraces *vs*. 97 cultivars) was used.

#### 3.3.2. Spatially: Genetic Diversity *vs*. Place of Origin

Generally speaking, great genetic variation should exist in the center of origin and domestication. Moreover, Vavilov reported that the Middle, Near East regions, and North Africa are considered the centers of origin and diversification of durum wheat [66]. However, in this present study, comparative analysis of genetic diversity among the 10 mega ecogeographical regions indicated that the greatest genetic diversity was found in South America, followed by North America and Western Europe, while Middle East showed moderate levels of genetic diversity (Table 6).

These results support the idea that the centers of diversity are not confined exclusively to their centers of origin [5,67]. Harlan [68,69] studied the distribution of variability in crops and concluded that there exist several centers of diversity in different crops which could not be regarded as centers of their origin. But it is worth pointing out that our results correspond to the centers of genetic diversity described by Vavilov [64]: North Africa should be considered as a microcenter of diversity for durum wheat in the southeastern Mediterranean (Table 6).

Higher genetic diversity in the New World than in the Old World where durum evolved was detected. The reason can be explained by a combination of the uneven distribution of landraces or cultivars among countries and different genetic diversity levels between landraces and cultivars used in this study. As shown in Table 2, the greatest genetic diversity was found in the cultivars released from PGR, followed by landraces, old cultivars, and EGR. In this study, a larger number of cultivars released during the period of 1981–2009 existed in ecogeographical regions having greater genetic diversity such as South America, North America, and Western Europe. For example, of the 33 accessions from North America, there are 24 cultivars released during the period of 1981–2009, accounting for 72.7%. To the contrary, Middle East has relatively lower genetic diversity based on 32 accessions, 18 of which are landraces, and 9 are old cultivars.

### 3.4. Divergence between Landraces and Cultivars Revealed by SNP Markers

Durum wheat had undergone intensive selection during domestication and the subsequent breeding process for certain desirable characteristics, such as high and stable yields. Such artificial selection activities may result in significant differentiation at some loci during domestication and the subsequent breeding process, since traits, e.g., grain yield, seed size, plant height, *etc*., are quantitatively inherited [1]. A *Fst*-outlier method was used to identify loci that may be under positive selection and therefore might be linked to genome regions conferring the phenotypic variation present in the analyzed germplasm.

We identified 92 candidate loci under positive selection based on *Fst* values that fall outside of the 99% confidence interval established for the distribution. These loci may be directly under selection, but more likely mark regions of the genome that have been selected during evolution. The loci we identified have a disproportional bias with 54.3% mapping to chromosomes 2, 6 and 7 (Figure 3, Table 5). This observation suggests that there are “hot spots” for directional selection in durum wheat. In addition, seven genes including P-EA, TsPAP1, CPK10, PI-PLC1, RSZ38, PDS, and LOX3, which play important roles in plant responses to biotic and abiotic stresses or in grain storage in wheat, appear to be under selection when comparing landraces with cultivars (Table 5). These results suggest that the use of objective approaches to identify outliers will reveal portions of the genome that are under selection. Such objective assessment will provide a scalable means for comprehensive assessments of genetic variation within durum wheat as emerging sequence data and improved genotyping platforms lead to larger data sets [49].

## 4. Experimental Section

### 4.1. Plant Materials

**Figure 4:**
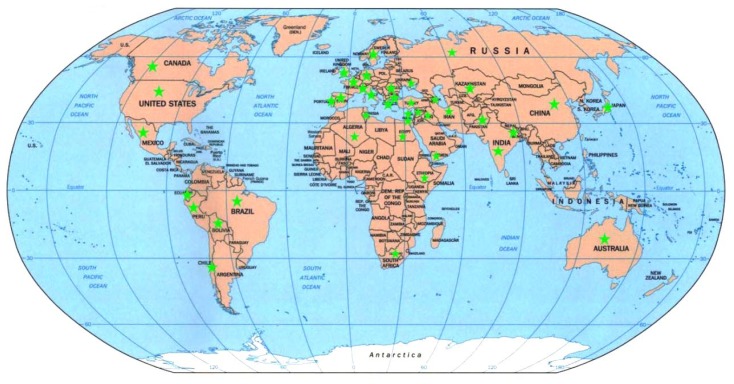
Geographical distribution of durum wheat accessions used in the present study. Only those countries with durum wheat sampling are indicated by green asterisks.

A total of 150 durum wheat accessions consisting of 53 landraces and 97 cultivars were used in this study. Ninety-seven cultivars were further divided into three temporal groups according to their released period: group 1, 1930–1964 (old cultivars, OC); group 2, 1965–1980 (Early Green Revolution, EGR); group 3, 1981–2009 (Post Green Revolution, PGR) [62,63,70,71]. The “Early Green Revolution” was characterized by breeding semi-dwarf varieties. The first semi-dwarf durum variety was released in Mexico in 1965 [60,61]. These 150 accessions were collected from 10 mega ecogeographical regions: East Asia, South Asia, Middle East, North America, South America, Oceania, Western Europe, Eastern Europe, South Africa, and North Africa, covering 41 countries and spatially reflecting different genetic backgrounds (Figure 4). Detailed information about each accession is shown in Table 7.

### 4.2. Genomic DNA Extraction and SNP Genotyping

Young leaves from each accession were collected and frozen in liquid nitrogen. Genomic DNA was isolated using a modified SDS (Sodium dodecyl sulfate) method according to Peng *et al*. [72]. The extraction buffer (pH 7.8–8.0) consisting of 500 mM sodium chloride (NaCl), 100 mM tris (hydroxymethyl) aminomethane hydrochloride (Tris–HCl) pH 8.0, 50 mM ethylene diamine tetraacetic acid (EDTA) pH 8.0, 0.84% (*w*/*v*) Sodium dodecyl sulfate (SDS), and 0.38% (*w*/*v*) sodium bisulfate.

The 150 durum wheat accessions were genotyped with 1536 SNP markers. These SNPs, discovered in a panel of 32 lines of tetraploid and hexaploid wheat, were downloaded from the Wheat SNP Database [73]. SNP selection and assay design were performed according to previously described procedures [35,74]. The following criteria were applied for SNP selection: no more than 2 SNPs were selected per locus, with preference being given to SNPs present in at least two lines in the discovery panel. Additional SNPs were discovered by sequencing the transcriptomes of *T. aestivum* cv. Chinese Spring and Jagger [35,74].

A total of 150 ng of genomic DNA per genotype was used for Illumina SNP genotyping at the Genome Center of University of California in Davis using Illumina Bead Array platform and Golden Gate Assay following the manufacturer’s protocol [75]. Genotype scores were called using the Illumina’s Genome Studio V 2010.3. Each of the 1536 SNP clusters was manually examined to correct imperfect calling of automated clustering.

### 4.3. Genetic Diversity

Genetic diversity was evaluated using POWERMARKER Ver. 3.25 [76]. The genetic parameters including Nei’s gene diversity and polymorphism information content (PIC) were used. Nei’s gene diversity was defined as the probability that two randomly chosen alleles from the population are different [77]. PIC values provide an estimate of the probability of finding polymorphism between two random samples of the germplasm.

### 4.4. Genetic Structure and Population Differentiation

In order to have a better insight into the genetic structure of durum wheat, different methods were exploited. First, we applied the Bayesian model-based clustering algorithm implemented in STRUCTURE 2.2 [78]. Admixture and correlated allele frequency models were employed with a number of clusters (K) ranging from 1 to 12. For each *K*, five runs were carried out. Burn-in time and replication number were both set to 100,000 for each run. Accessions with probability of membership greater than 80% were assigned to a subgroup, while those with lower probabilities were assigned to the “mixed” subgroup. Dendrograms, based on the NJ algorithm according to shared-allele distance, were also used to analyze the genetic structure of the germplasm. A phylogenetic tree was implemented by POWERMARKER Ver. 3.25. Bootstrapping over loci with 1000 replications was carried out to assess the strength of the evidence for the branching patterns in the resulting NJ tree. A consensus tree with bootstrap values was reconstructed by the consensus program of PHYLIP [79] and displayed by FigTree Ver.1.3.1[80].

The population differentiation was assessed with the AMOVA implemented in the ARLEQUIN version 3.11software [81]. Significance levels for variance components were estimated using 16,000 permutations. We identified loci under positive selection between landrace and cultivars using a *Fst*-outlier detection method as implemented in the LOSITAN workbench [50]. The analysis was performed with 100,000 simulations using an infinite allele model. Based on *Fst* values that fall outside of the 99% confidence interval, candidate loci identified under positive selection were used for further analysis.

### 4.5. Statistical Tests

SPSS V.13.0 program was used for statistical analyses [82]. The significance of differences for Nei’s gene diversity and PIC among chromosomes was tested by estimating a 95% confidence interval (CI) of the genome mean, which was calculated using bootstrap analysis with 1000 replications. Chromosome means outside of the 95% CI were declared significantly different from the genome mean [36]. The Paired *t* test was used to test the significance of differences of genetic diversity between genomes using Nei’s gene diversity and PIC per chromosome as variables. The significance of differences for genetic diversity parameters between cultivars and landrace were also tested by paired *t* test. The plant height data were analyzed by analysis of variance (ANOVA) and the means among group were further tested by Duncan’s Multiple Range Test.

## 5. Conclusions

In this study, we used worldwide germplasm accessions and 946 SNP markers to estimate genetic structure and genetic diversity of durum wheat on the whole genome level. Genetic structure, based on a set 150 accessions from different places of origin, was greatly affected by many factors, such as environmental conditions, release period of varieties, and gene flows via germplasm exchanges or human activities. Genetic diversity indicated that there still existed a substantial level of genetic variation within modern cultivars of durum wheat as detected by SNP markers, despite rigorous selection pressure aimed at cultivar purity and associated breeding practices. Our results can be used to accelerate wheat improvement by addressing the patterns of genetic variation within durum wheat, conserving adequate type and number of germplasm accessions and helping breeders maximize the level of variation present in segregating populations by crossing cultivars with greater genetic distance.

## Figures and Tables

**Table 1:** Distribution and diversity index of 946 single nucleotide polymorphism (SNP) markers in a set of 150 *T. durum* accessions.

Chromosome	No. of SNP Markers	No. of Polymorphic Markers	Gene Diversity	PIC
**A Genome**				
1A	114	75	0.2319	0.1905
2A	96	65	0.2180	0.1840
3A	98	67	0.2036	0.1697
4A	124	86	0.1899 *	0.1576 *
5A	85	59	0.2179	0.1798
6A	125	78	0.2526 *	0.2072 *
7A	135	88	0.2249	0.1884

Subtotal/Mean	767	516	0.2193	0.1819

**B Genome**				
1B	99	76	0.2695 *	0.2225 *
2B	87	64	0.2553	0.2097
3B	67	49	0.2180 *	0.1832
4B	75	46	0.2200 *	0.1804 *
5B	76	49	0.2120 *	0.1747 *
6B	105	83	0.2211 *	0.1842
7B	101	70	0.2404	0.1982

Subtotal/Mean	599	430	0.2384	0.1970

**Hemoeologous**				
1	213	151	0.2508 *	0.2066 *
2	183	129	0.2365	0.1967
3	165	116	0.2097 *	0.1754 *
4	199	132	0.2004 *	0.1656 *
5	161	108	0.2153 *	0.1775 *
6	230	161	0.2364	0.1953
7	236	158	0.2318	0.1927

Total/Grand mean	1366	946	0.2280	0.1888

* Means outside of the 95% bootstrap confidence interval of the genome mean.

**Table 2:** Comparison of genetic diversity generated by 946 SNP markers between landraces and cultivars.

	Sample Size	No. of Polymorphic Marker	Polymorphic Rate (%)	Gene Diversity *	PIC *
**Improvement status**					
Landrace	53	756	79.9%	0.2192 b	0.1800 b
Cultivar	97	933	98.6%	0.2310 a	0.1919 a

**Time group**†					
Landrace	53	756	79.9%	0.2192 b	0.1800 b
OC	32	757	80.0%	0.2192 b	0.1807 b
EGR	35	728	77.0%	0.2034 c	0.1680 c
PGR	30	825	87.2%	0.2474 a	0.2039 a

† OC-old cultivars released before 1965; EGR, cultivars released during the period of early Green Revolution (1965–1980); PGR, cultivars released during the period of post Green Revolution (1981–2009).

* Significance was tested by the paired *t* test and means in each column followed by same letters was indicated by different letters at *p* ≤ 0.05.

**Table 3:** Analysis of molecular variance (AMOVA) between landraces and cultivars.

Source of Variation	Sum of Squares	Percentage of Variation (%)
Among Populations	321.84	0.50
Within Population (Cultivar)	42,400.65	65.54
Within Population (landrace)	21,977.11	33.97
Total	64,699.60	100.00

**Table 4:** Plant height of various group of durum wheat germplasm.

Group	Sample Size	Mean Plant Height, cm (SE)
Landrace	53	132.46 (1.91) a
OC	32	130.72 (2.48) a
EGR	35	119.13 (4.05) b
PGR	30	101.91 (4.27) c

Means in each column followed by same letters are not significantly different at *p* ≤ 0.05 as determined by Duncan’s Multiple Range Test; OC, old cultivars released before 1965; EGR, cultivars released during the period of early Green Revolution (1965–1980); PGR, cultivars released during the period of post Green Revolution (1981–2009).

**Table 5:** ESTs and the plausible functions in the homologous ESTs outlier loci between landrace and cultivar.

SNP marker and the EST				Gene function and the homologous EST			
	
Code	SNP Marker	Accession No.	Map position (Bin)	Function	Accession No.	Identity (%)	*E*-value
Outlier 1	AY244508_5_B_Y_26	AY244508	5B	G1777 MADS-box transcriptional factor (AP1) gene, *T. monococcum*	AY244508.1		
Outlier 2	BE405518_1_A_95	BE405518	1AS3-0.86–1.00	Alternative splicing regulator (RSZ38), *T. aestivum*	DQ019628.1	93%	0
Outlier 3	BE405518_1_A_Y_106	BE405518	1AS3-0.86–1.00	Alternative splicing regulator (RSZ38), *T. aestivum*	DQ019628.1	93%	0
Outlier 4	BE442666_4_A_269	BE442666	4AL13-0.59–0.66	Lipoxygenase 3 (LOX3), *T. aestivum*	HQ913602.1	99%	0
Outlier 5	BE442666_4_B_Y_327	BE442666	4BS8-0.57–0.81	Lipoxygenase 3 (LOX3), *T. aestivum*	HQ913602.1	99%	0
Outlier 6	BE404341_5_B_Y_124	BE404341	5B	Phytochelatin synthetase, *T. aestivum*	AY442329.1	98%	0
Outlier 7	BE406148_7_B_Y_647	BE406148	7BL7-0.63–0.78	Cyclophilin B-B gene, *T. aestivum*	EU627095.1	100%	9 × 10^−101^
Outlier 8	BE445506_7_B_Y_355	BE445506	7BL10-0.78–1.00	Unknown			
Outlier 9	BE405834_1_A_N_641	BE405834	1AS3-0.86–1.00	Soluble inorganic pyrophosphatase-like, *B. distachyon*	XM_003568957.1	91%	0
Outlier 10	BE405834_1_B_Y_216	BE405834	1BL1-0.47–0.69	Soluble inorganic pyrophosphatase-like, *B. distachyon*	XM_003568957.1	91%	0
Outlier 11	BE446240_1_B_131	BE446240	1BL1-0.47–0.69	Rab GDP dissociation inhibitor, *B. distachyon*	XM_003568390.1	93%	0
Outlier 12	BE403177_2_B_409	BE403177	2B	F-box protein 7-like, *B. distachyon*	XM_003579715.1	90%	3 × 10^−136^
Outlier 13	BE404332_2_B_29	BE404332	C-2BS4-0.75 *	Ribosomal protein S12 (rps12), *H. vulgare*	AF067732.1	94%	0
Outlier 14	BE444144_2_B_92	BE444144	2BS	Unknown			
Outlier 15	BE445278_2_B_143	BE445278	2B	RuvB-like 2-like, *B. distachyon*	XM_003562775.1	92%	0
Outlier 16	BE445278_2_B_243	BE445278	2B	RuvB-like 3-like, *B. distachyon*	XM_003562775.1	92%	0
Outlier 17	BE445242_2_A_362	BE445242	C-2AS5-0.78	Unknown			
Outlier 18	BE444579_3_B_Y_375	BE444579	3B	Unknown			
Outlier 19	BE444864_3_B_373	BE444864	3BL7-0.63–1.00	C2 domain-containing protein C31G5.15-like, *B. distachyon*	XR_138068.1	91%	0
Outlier 20	BE443187_5_A_511	BE443187	5AL12-0.35–0.57	65-kDa microtubule-associated protein 7-like, *B. distachyon*	XM_003578156.1	88%	0
Outlier 21	CD373602_5_B_Y_310	CD373602	5BL16-0.79–1.00	Unknown			
Outlier 22	BE444256_6_A_N_1118	BE444256	C-6AL4-0.55	Alcohol dehydrogenase-like 6-like*, B. distachyon*	XM_003569903.1	93%	0
Outlier 23	CD452643_6_B_111	CD452643	6BL3	Alcohol dehydrogenase-like 6-like, *B. distachyon*	XM_003569903.1	92%	1 × 10^−117^
Outlier 24	CD452643_6_B_Y_113	CD452643	6BL3	Alcohol dehydrogenase-like 6-like, *B. distachyon*	XM_003569903.1	92%	1 × 10^−117^
Outlier 25	BE446380_7_A_577	BE446380	7AS8-0.45–0.59	Putative phospholipid-transporting ATPase 9-like, *B. distachyon*	XM_003563827.1	91%	0
Outlier 26	BE403950_6_B_Y_325	BE403950	6BL5-0.40–1.00	ABC transporter F family member 3-like, *B. distachyon*	XM_003570443.1	93%	0
Outlier 27	BE517729_1_A_116	BE517729	1AL3-0.61–1.00	Putative prolyl aminopeptidase 1 (PAP1), T. durum × Secalecereale	JN808306.2	97%	0
Outlier 28	BE517729_1_A_Y_117	BE517729	1AL3-0.61–1.00	Putative prolyl aminopeptidase 1 (PAP1), T. durum × Secalecereale	JN808306.2	97%	0
**Outlier 29**	BE517831_2_B_70	BE517831	C-2BL2-0.36	Phosphoinositide-specific phospholipase C1, *T. aestivum*	HM754654.1	95%	0
**Outlier 30**	BF200531_1_A_N_573	BF200531	1AS3-0.86–1.00	Protein notum homolog, *B. distachyon*	XM_003566643.1	94%	4 × 10^−169^
**Outlier 31**	BF474493_6_A_N_40	BF474493	C-6AL4-0.55	Pescadillo homolog, *B. distachyon*	XM_003560899.1	91%	0
**Outlier 32**	BF474139_1_A_144	BF474139	1AL3-0.61–1.00	6 phosphofructo kinase 3-like*, B. distachyon*	XM_003568020.1	95%	6 × 10^−157^
**Outlier 33**	BF201102_5_B_444	BF201102	5BS6-0.81–1.00	Methionine synthase 1 enzyme (ms1 gene), *Hordeum vulgare*	AM039904.1	93%	2 × 10^−168^
**Outlier 34**	BF201102_5_B_Y_373	BF201102	5BS6-0.81–1.00	Methionine synthase 1 enzyme (ms1 gene), *Hordeum vulgare*	AM039904.1	93%	2 × 10^−168^
**Outlier 35**	CD453605_6_B_427	CD453605	6B	Putative nitric oxide synthase-like, *B. distachyon*	XM_003570728.1	89%	2 × 10^−179^
**Outlier 36**	BF474379_7_A_83	BF474379	7AL16-0.86–0.90	Protein N-terminal asparagine amidohydrolase-like, *B. distachyon*	XM_003563571.1	90%	0
**Outlier 37**	BF474379_7_A_Y_253	BF474379	7AL16-0.86–0.90	Protein N-terminal asparagine amidohydrolase-like, *B. distachyon*	XM_003563571.1	90%	0
**Outlier 38**	BE494527_1_B_77	BE494527	1BL2-0.0.69–0.85	Phosphoethanolamine methyltransferase, *T. aestivum*	AY065971.1	96%	3 × 10^−86^
**Outlier 39**	BE494527_1_B_Y_438	BE494527	1BL2-0.0.69–0.85	Phosphoethanolamine methyltransferase, *T. aestivum*	AY065971.1	96%	3 × 10^−86^
**Outlier 40**	BE494765_4_B_Y_426	BE494765	4BL5-0.86–1.00	Unknown			
**Outlier 41**	BE636872_6_A_119	BE636872	6A	Unknown			
**Outlier 42**	BE495277_5_B_336	BE495277	C-5BL14-0.75 *	UPF0664 stress-induced protein C29B12.11c-like, *B. distachyon*	XM_003578371.1	91%	2 × 10^−137^
**Outlier 43**	BE493868_7_A_Y_93	BE493868	7AS5-0.59–0.89	Probable protein phosphatase 2C 54-like, *B. distachyon*	XM_003564166.1	91%	0
**Outlier 44**	BE494482_7_B_Y_29	BE494482	7B	Zuxin response factor 21 (ARF21) gene, *Zea mays*	HM004536.1	92%	3 × 10^−67^
**Outlier 45**	CD491758_6_A_Y_81	CD491758	6A	Calcium-dependent protein kinase-like (CPK10), *T. aestivum*	EU181189.1	92%	0
**Outlier 46**	BQ159615_6_B_Y_336	BQ159615	6B	Leucine-rich repeat protein (LRR2), *T. aestivum*	EF555120.1	98%	0
**Outlier 47**	BF291774_6_B_181	BF291774	6BSc	Putative vacuolar cation/proton exchanger 4-like, *B. distachyon*	XM_003570864.1	83%	0
**Outlier 48**	BF292264_7_A_712	BF292264	7AS1-0.89–1.00	Unknown			
**Outlier 49**	BF292193_7_B_N_78	BF292193	7BL7-0.63–0.78	Cytochrome b5 (cb5-1 gene), *Oryza sativa*	AJ429043.1	84%	8 × 10^−103^
**Outlier 50**	BF291774_6_B_519	BF291774	6BSc	Putative vacuolar cation/proton exchanger 4-like, *B. distachyon*	XM_003570864.1	83%	0
**Outlier 51**	BG263233_1_B_825	BG263233	1BL2-0.0.69–0.85	Flap endonuclease 1-A-like, B. distachyon	XM_003567949.1	91%	0
**Outlier 52**	BG605368_2_A_156	BG605368	C-2AL1-0.85	Exopolygalacturonase-like, *B. distachyon*	XM_003571584.1	86%	4 × 10^−136^
**Outlier 53**	BG605368_2_A_Y_310	BG605368	C-2AL1-0.85	Exopolygalacturonase-like, *B. distachyon*	XM_003571584.1	86%	4 × 10^−136^
**Outlier 54**	BG263521_2_B_Y_261	BG263521	C-2BS1-0.53	Mitogen activated protein kinase (MEK1), *O, sativa*	AF080436.1	83%	4 × 10^−141^
**Outlier 55**	BF203070_3_B_Y_52	BF203070	3BS9-0.57–0.78	Unknown			
**Outlier 56**	BE637808_4_A_Y_332	BE637808	4A	DEAD-box ATP-dependent RNA helicase 16-like, *B. distachyon*	XM_003559423.1	90%	4 × 10^−165^
**Outlier 57**	BF482950_4_A_Y_272	BF482950	4A	Lariat debranching enzyme-like, *B. distachyon*	XM_003559432.1	90%	7 × 10^−117^
**Outlier 58**	BF483551_4_A_N_203	BF483551	4AS3-0.76–1.00	Unknown			
**Outlier 59**	BE497820_5_A_Y_664	BE497820	C-5AL10-0.57 *	Probable thylakoidal processing peptidase 2, chloroplastic-like, *B. distachyon*	XM_003578166.1	89%	0
**Outlier 60**	BE498662_7_A_Y_513	BE498662	7AS8-0.45–0.59	Unknown			
**Outlier 61**	BF482403_7_A_126	BF482403	7AL21-0.74–0.86	Unknown			
**Outlier 62**	BQ169669_7_A_Y_378	BQ169669	7AL18	Unknown			
**Outlier 63**	BE499248_7_B_Y_63	BE499248	7BS1-0.27–1.00	Caffeoyl-CoA O-methyltransferase 2, *B. distachyon*	XM_003564219.1	95%	6 × 10^−153^
**Outlier 64**	BF485380_7_B_Y_479	BF485380	7B	Unknown			
**Outlier 65**	BM140362_1_B_432	BM140362	1BL1-0.47–0.69	Glyoxysomal processing protease, glyoxysomal-like, *B. distachyon*	XM_003568135.1	89%	0
**Outlier 66**	BG604678_4_A_Y_256	BG604678	4AL13-0.59–0.66	Phytanoyl-CoA dioxygenase domain-containing protein 1-like*, B. distachyon*	XM_003560712.1	92%	0
**Outlier 67**	CD453913_7_A_105	CD453913	7A	Phosphoserine phosphatase, chloroplastic-like, *B. distachyon*	XM_003577403.1	89%	2 × 10^−179^
**Outlier 68**	BG262421_6_A_87	BG262421	6AS1-0.35–0.65	Purple acid phosphatase 18-like, *B. distachyon*	XM_003562305.1	91%	0
**Outlier 69**	BG262287_7_B_Y_175	BG262287	7B	Vacuolar proton-ATPase subunit A, *T. aestivum*	DQ432014.1	99%	0
**Outlier 70**	BE490763_2_A_1462	BE490763	2AL1-0.85–1.00	Endoplasmic reticulum metallopeptidase 1-like, *B. distachyon*	XM_003580100.1	88%	0
**Outlier 71**	BE471213_6_A_N_28	BE471213	6AL8-0.90–1.00	Metal tolerance protein C2-like, *B. distachyon*	XM_003570688.1	92%	6 × 10^−178^
**Outlier 72**	BE591172_4_B_Y_148	BE591172	4BL5-0.86–1.00	Phytoenedesaturase (PDS), *T. aestivum*	FJ517553.1	98%	0
**Outlier 73**	BE591974_5_A_1534	BE591974	5AS1-0.40–0.75	Unknown			
**Outlier 74**	BE591290_1_B_Y_289	BE591290	1BL2-0.0.69–0.85	B73 WTF1 gene, Zea mays cultivar	FJ264201.1	82%	2 × 10^−134^
**Outlier 75**	BE591002_7_A_244	BE591002	7AL17-0.71–0.74	Probable alanyl-t RNA synthetase, chloroplastic-like, transcript variant 2, *B. distachyon*	XM_003563964.1	85%	2 × 10^−108^
**Outlier 76**	BE591777_6_A_Y_394	BE591777	6AL8-0.90–1.00	PAP-specific phosphatase HAL2-like, *B. distachyon*	XM_003570307.1	89%	1 × 10^−128^
**Outlier 77**	BE497494_2_A_Y_475	BE497494	2AS5-0.78–1.00	GLU gene for ferredoxin-dependent glutamate synthase precursor, *O. sativa*	AB061357.1	96%	0
**Outlier 78**	BE497224_4_A_Y_41	BE497224	4AS1-0.20–0.63	Unknown			
**Outlier 79**	BE605194_7_B_Y_583	BE605194	7BL10-0.78–1.00	Serine/threonine-protein kinase At5g01020-like, *B. distachyon*	XM_003563310.1	92%	2 × 10^−131^
Outlier 80	BG275030_2_A_96	BG275030	2AS5-0.78–1.00	Symplekin-like, *B. distachyon*	XM_003559695.1	91%	4 × 10^−144^
Outlier 81	BG275030_2_A_Y_103	BG275030	2AS5-0.78–1.00	Symplekin-like, *B. distachyon*	XM_003559695.1	91%	4 × 10^−144^
Outlier 82	BF475120_6_B_Y_75	BF475120	6BL5-0.40–1.00	Unknown			
Outlier 83	BG313707_5_A_Y_547	BG313707	5AS1-0.40–0.75	2 oxoglutarate/malate translocator, chloroplastic-like, *B. distachyon*	XM_003575906.1	93%	3 × 10^−160^
Outlier 84	BG314532_2_A_Y_446	BG314532	2AS5-0.78–1.00	Unknown			
Outlier 85	BQ168780_5_B_995	BQ168780	C-5BL14–0.75 *	Actin-related protein 2/3 complex subunit 5-like, *B. distachyon*	XM_003577407.1	92%	1 × 10^−145^
Outlier 86	BG314551_3_A_Y_162	BG314551	3AS4-0.45–1.00	66 kDa stress protein-like, *B. distachyon*	XM_003567837.1	87%	4 × 10^−176^
Outlier 87	BQ168329_2_A_Y_198	BQ168329	2A	Protoporphyrin IX Mg-chelatase subunit precursor (Xantha-f) gene, *H. vulgare*	U26916.1	97%	0
Outlier 88	BE426222_3_A_68	BE426222	C-3AS2-0.23	Topless-related protein 2-like, transcript variant 1, *B. distachyon*	XM_003566383.1	91%	0
Outlier 89	BE489326_3_B_Y_300	BE489326	C-3BL2-0.22	CTD-phosphatase-like protein, *Zea mays*	NM_001155943.1	80%	1 × 10^−115^
Outlier 90	BE425301_4_A_Y_160	BE425301	4AS4-0.63–0.76	40S ribosomal protein gene, *T. aestivum*	AF479043.1	99	5 × 10^−175^
Outlier 91	BE426413_6_B_286	BE426413	C-6BL5-0.40 *	Adenosine kinase 2-like, *B. distachyon*	XM_003575347.1	94%	0
Outlier 92	BJ291318_5_B_Y_120	BJ291318	5B	60S ribosomal protein L23a-like, *B. distachyon*	XM_003557882.1	87%	2 × 10^−179^

**Table 6:** SNP-based genetic diversity generated by 946 SNP markers in durum wheat from 10 mega ecogeographic origins.

Origin	Sample Size	Gene Diversity	PIC
East-Asia	15	0.2220	0.1798
Eastern-Europe	15	0.2183	0.1792
Latin-America	12	0.2518	0.2044
Middle-East	32	0.1906	0.1549
North-Africa	12	0.2054	0.1682
North-America	33	0.2351	0.1937
Oceania	7	0.2179	0.1747
South-Africa	4	0.1591	0.1252
South-Asia	6	0.1575	0.1258
Western-Europe	14	0.2299	0.1902

**Table 7:** List of durum wheat accessions used in the study. Geographical region of origin, year of release, accession identifier #, geographical parameters, and improvement status are reported.

Geographical Region of Origin	Country	Region within Country	Code	Accession Identifier#	Collection Year	Latitude	Longitude	Elevation
**East Asia (15)**	China	Heilongjiang	PDW1	CItr 11495	1932	48.00N	128.00E	
		Heilongjiang	PDW238 ^*^	PI 70658	1926	45.75N	126.65E	140
		Heilongjiang	PDW239 ^*^	PI 70662	1926	45.76N	126.66E	140
		Heilongjiang	PDW245 ^*^	PI 79900	1929			
		Xinjiang	PDW161	PI 447421	1980			
		Jiangsu	PDW40 ^*^	PI 124292	1937	31.75N	120.25E	
		Jiangsu	PDW244 ^*^	PI 74830	1927	33N	120E	
		Beijing	PDW27 ^*^	CItr 5094	1916	39.93N	116.40E	62
		Sichuan	PDW31 ^*^	CItr 8327	1924	28.83N	104.58E	452
		unknown	PDW25 ^*^	CItr 5077	1916			
		unknown	PDW26 ^*^	CItr 5083	1916			
		unknown	PDW85	PI 283853	1962			
		unknown	PDW159	PI 435100	1979			
	Japan	Hokkaido	PDW222 ^*^	PI 61351	1924	40.71N	142.50E	
		Hokkaido	PDW223 ^*^	PI 61352	1924	40.72N	142.51E	

**Central Asia (2)**	Kazakhstan	Kazakhstan	PDW217 ^*^	PI 61112	1924	50.47N	80.22E	220
		Kazakhstan	PDW218 ^*^	PI 61123	1924	50.48N	80.23E	220

**South Asia (6)**	Nepal	Sonsera	PDW51 ^*^	PI 176228	1949			2128
	Pakistan	Punjab	PDW64	PI 210910	1953	31.00N	72.00E	
		Punjab	PDW65	PI 210911	1953	31.01N	72.01E	
		Punjab	PDW142 ^*^	PI 388132	1974	31.02N	72.02E	
	India	Madhya Pradesh,	PDW145 ^*^	PI 41015	1915	22.00N	79.00E	
		Gujarat	PDW146 ^*^	PI 41342	1915	21.70N	72.97E	

**Middle East (32)**	Turkey	Ankara	PDW36	PI 109588	1935	39.53N	32.63E	938
		Bitlis	PDW192 ^*^	PI 560717	1986	38.77N	42.37E	1770
		Bitlis	PDW193 ^*^	PI 560718	1986	38.78N	42.38E	1770
		Siirt	PDW190 ^*^	PI 560702	1986	37.82N	41.87E	560
		Siirt	PDW194 ^*^	PI 560889	1989	37.75N	42.20E	1070
		unknown	PDW102	PI 346985	1970			
	Syria	Dimashq	PDW52 ^*^	PI 182697	1949	33.5N	36.30E	690
		Halab	PDW57 ^*^	PI 193391	1951	36.2N	37.17E	410
		Unknown	PDW180	PI 520415	1987			
		Unknown	PDW41 ^*^	PI 134596	1939			
	Iran	Khuzestan,	PDW42 ^*^	PI 140184	1941	32.38N	48.40E	126
		East Azerbaijan	PDW72 ^*^	PI 222675	1954	38.08N	46.30E	1399
		Tehran	PDW76 ^*^	PI 243790	1957	35.27N	49.28E	1866
		Fars	PDW88 ^*^	PI 289821	1963	30.33N	51.52E	1130
	Iraq	Ninawa	PDW79 ^*^	PI 253801	1958	36.33N	43.13E	223
		Unknown	PDW47	PI 165846	1948			
		Unknown	PDW58 ^*^	PI 208903	1953			
		Unknown	PDW60 ^*^	PI 208907	1953			
		Unknown	PDW61 ^*^	PI 208908	1953			
		Unknown	PDW62 ^*^	PI 208910	1953			
		Unknown	PDW242 ^*^	PI 70736	1926			
	Israel	Unknown	PDW77	PI 249816	1958			
		Unknown	PDW78	PI 249820	1958			
		Unknown	PDW90	PI 292035	1963			
		Unknown	PDW139	PI 384043	1973			
		Unknown	PDW141	PI 388035	1974			
	Cyprus	Unknown	PDW68 ^*^	PI 210952	1953			
		Unknown	PDW75	PI 237632	1957			
		Unknown	PDW208	PI 591959	1994			
	Yemen	Aden	PDW45	PI 152567	1945	12.77N	45.01E	79
	Azerbaijan	Unknown	PDW73	PI 233213	1956			
		Unknown	PDW101	PI 345707	1950			

**North America (33)**	USA	North Dakota	PDW3	Citr 12068	1940			
		North Dakota	PDW7	Citr 13246	1955			
		North Dakota	PDW8	Citr 13333	1957			
		North Dakota	PDW288	Ldn 16				
		Colorado	PDW29	Citr 6881	1923			
		Kansas	PDW189	PI 560335	1992			
		Arizona	PDW200	PI 573005	1988			
		Arizona	PDW211	PI 601250	1985			
		California	PDW210	PI 600931	1982			
		California	PDW231	PI 656793	2009			
		California	PDW232	PI 656794	2009			
		California	PDW233	PI 656795	2009			
		Erevan	PDW250	PI 9872	1903	40.18N	44.50E	1120
	Mexico	Federal District	PDW152	PI 428453	1978			
		Federal District	PDW173	PI 519751	1987			
		Federal District	PDW174	PI 519752	1987			
		Federal District	PDW176	PI 519761	1987			
		Federal District	PDW177	PI 519866	1987			
		Federal District	PDW178	PI 520053	1987			
		Federal District	PDW216	PI 610765	1999			
		Federal District	PDW227	PI 634315	2001			
		Federal District	PDW229	PI 634318	2001			
		Unknown	PDW179	PI 520173	1987			
		Unknown	PDW49	PI 168708	1948			
		Unknown	PDW150	PI 422289	1978			
		Unknown	PDW13	Citr 15874	1972			
	Canada	Saskatchewan	PDW18	Citr 17337	1974			
		Saskatchewan	PDW186	PI 546060	1990			
		Saskatchewan	PDW187	PI 546362	1991			
		Saskatchewan	PDW202	PI 583724	1994			
		Saskatchewan	PDW205	PI 583731	1994			
		Saskatchewan	PDW206	PI 583732	1994			
		Saskatchewan	PDW207	PI 583733	1994			

**Latin America (12)**	Chile	La Araucania	PDW14	Citr 17057	1972			
		La Araucania	PDW15	Citr 17058	1972			
		La Araucania	PDW16	Citr 17157	1972			
		La Araucania	PDW17	Citr 17159	1972			
	Peru	Junin	PDW248	PI 91956	1931	12.03S	75.28W	3252
		Cajamarca	PDW249	PI 92024	1931	7.60S	78.47W	3050
		Unknown	PDW48	PI 168692	1948			
	Brazil	Sao Paulo	PDW54	PI 191645	1950	22.00S	49.00W	
		Unknown	PDW175	PI 519759	1987			
	Bolivia	Cochabamba	PDW196 ^*^	PI 565259	1991	17.40S	66.23W	3245
		Cochabamba	PDW197 ^*^	PI 565266	1991	17.57S	65.83W	2730
	Ecuador	Pichincha	PDW87	PI 286546	1963			

**Oceania (7)**	Australia	Victoria	PDW28 ^*^	Citr 5136	1916	34.25S	141.50E	
		Western Australia	PDW50	PI 174645	1949			
		Western Australia	PDW235	PI 67341	1926			
		New South Wales	PDW74	PI 235159	1956	33.00S	146.00E	
		Unknown	PDW34	PI 107606	1934			
		Unknown	PDW138	PI 377882	1973			
		Unknown	PDW153	PI 428701	1978			

**Western Europe (14)**	Portugal	Lisboa	PDW195	PI 56233	1923			
	France	Unknown	PDW124	PI 352450	1969			
	Greece	Unknown	PDW106	PI 352389	1969			
	Sweden	Gotland	PDW56	PI 192711	1950			
	Switzerland	Switzerland	PDW105	PI 352377	1969			
	Spain	Unknown	PDW112	PI 352404	1969			
	Germany	Unknown	PDW22 ^*^	Citr 2468	1904			
	Germany	Lower Saxony	PDW93	PI 306664	1965			
	Bulgaria	Unknown	PDW100	PI 344743	1969			
	Bulgaria	Khaskovo	PDW188	PI 546462	1990			
	Italy	Unknown	PDW113	PI 352408	1969			
		Latium	PDW115	PI 352415	1969			
		Latium	PDW209	PI 593005	1996			
	England	Unknown	PDW83	PI 278223	1962			
		Unknown	PDW84	PI 278648	1962	53.00N	2.00W	
		Unknown	PDW95	PI 321702	1967			
	Romania	Unknown	PDW131	PI 376498	1972			
		Unknown	PDW132	PI 376500	1972			
		Unknown	PDW133	PI 376501	1972			
		Unknown	PDW135	PI 376509	1972			
		Unknown	PDW136	PI 376511	1972			
		Unknown	PDW137	PI 376512	1972			

**Eastern Europe (5)**	Ukraine	Kharkiv	PDW160	PI 438973	1980			
	Russian	Altay	PDW24 ^*^	Citr 3267	1911	52.68N	83.21E	152
		Former Soviet	PDW118	PI 352436	1969			
		Union						
		Former Soviet	PDW119	PI 352437	1969			
		Union						
		Krasnoyarsk	PDW220 ^*^	PI 61189	1924	58.45N	92.17E	79

**South Africa (4)**	South Africa	Unknown	PDW151 ^*^	PI 42425	1916			
		Free State	PDW163 ^*^	PI 45442	1917	29.17S	24.75E	1123
		Cape Province	PDW164 ^*^	PI 45443	1917	30.98S	27.33E	1703
		Cape Province	PDW167	PI 46766	1918	31.47S	19.78E	994

**North Africa (12)**	Algeria	Mascara	PDW39 ^*^	PI 11715	1904	35.74N	0.55E	104
	Tunisia	Unknown	PDW107	PI 352390	1969			
		Unknown	PDW170 ^*^	PI 51210	1920	33.02N	35.57E	
		Unknown	PDW171	PI 519380	1987			
	Egypt	Giza	PDW46	PI 153774	1946	29.77N	31.30E	
		Minufiya	PDW183	PI 532119	1988	30.47N	30.93E	12
		Unknown	PDW212 ^*^	PI 60712	1924			
		Sinai	PDW215 ^*^	PI 60742	1924	29.50N	34.00E	
		Alexandria	PDW237 ^*^	PI 7016	1901	31.17N	29.87E	
		Sawhaj	PDW243 ^*^	PI 7422	1901	26.35N	31.89E	65
	Ethiopia	Unknown	PDW110	PI 352395	1969			
		Unknown	PDW128 ^*^	PI 352551	1969			

Note: Accessions marked by ^*^ are landraces.
